# Synthesis and Anticancer Activity of Rhopaladins’ Analog RPDPD Against the HeLa Human Cervical Cancer Cell Line

**DOI:** 10.3389/fchem.2022.921276

**Published:** 2022-06-27

**Authors:** Feng Chen, Hong-Mei Wang, Ling-Qi Kong, Qin-Hua Chen, Li-Na Ke, He-Liu Dai, Xiao-Hua Zeng

**Affiliations:** ^1^ Sinopharm Dongfeng General Hospital, Hubei University of Medicine, Shiyan, China; ^2^ Hubei Key Laboratory of Wudang Local Chinese Medicine Research, School of Pharmaceutical Sciences, Hubei University of Medicine, Shiyan, China; ^3^ Shenzhen Baoan Authentic TCM Therapy Hospital, Shenzhen, China

**Keywords:** rhopaladins’ analog, synthesis, 2-aroyl-4-arylidene-5-oxopyrrolidine, cytotoxicity evaluation, HPV E6/E7 mRNA, TIMP3/MMP3 pathway

## Abstract

Heterocyclic compounds were widely used in many domains; pyrrolidone is a derivative of heterocycles that can be used to synthesize anticancer drugs. A new fluorine-containing rhopaladins’ analog(*E*)-2-(4-bromobenzoyl)-*N*-(tert-butyl)-4-(4-fluoro benzylidene)-5-oxo-1-propylpyrrolidine-2-carboxamide (RPDPD for short) of 2-aroyl-4-arylidene-5-oxopyrrolidine derivative was synthesized by the one-pot synthesis method and evaluated for its anti-tumor activity *in vitro via* CCK8 assay and annexin V/propidium iodide (PI) staining of HeLa cells. The results exhibited that compound RPDPD has inhibited the proliferation of HeLa in a dose-dependent manner with an IC_50_ of 24.23 μmol/L (*p* < 0.05) and has low hepatotoxicity with an IC_50_ of 235.6 μmol/L (*p* < 0.05) to normal hepatocyte LO2 cells. The apoptotic assay demonstrated that compound RPDPD has induced apoptosis in HeLa cells (from 14.26 to 23.4%, *p* < 0.05). qRT-PCR results showed that the compound RPDPD could inhibit the expression of oncogene E6/E7 mRNA (*p* < 0.05) of human papillomavirus (HPV). The results of Western blot showed that the compound RPDPD promoted the expression of TIMP3 protein and inhibited the expression of MMP3 (*p* < 0.05). In conclusion, the compound RPDPD can inhibit the proliferation of cervical cancer cells and induce the apoptosis of cervical cancer cells, and its mechanism may be related to the inhibition of E6 mRNA and E7 mRNA expressions, and the anticancer effect of the compound RPDPD on cervical cancer is closely related to the TIMP3/MMP3 signaling axis.

## Introduction

Heterocyclic compounds are extensively used in plenty of fields and are favored by many drug researchers. Pyrrolidone, one of the heterocyclic compounds, can be used as an antiviral (Nilsson et al., 1990), antiepileptic (Kenda et al., 2004), and anti-HIV (Kraus et al., 2000) agent. It has been reported that pyrrolidone can be used as an inhibitor of ceramide (Atigadda et al., 1999) and a selective inhibitor of matrix metalloproteinase (Wu and Feldkamp, 1961).

The incidence and development of cancer seriously threaten human health. The incidence rate of cervical cancer ranks fourth among cancer in women. In 2018, the incidence of new cases was about 570,000, and the death toll was about 310,000 for cervical cancer in the world (Freddie et al., 2018). The pathogenic factor of cervical cancer is human papillomavirus (HPV) (Ijang et al., 2019). HPV is a small enveloped DNA virus, which can be divided into two kinds of “low risk” or “high risk”, with the inherent disorder area; it is the most common route of transmission for sexually transmitted disease (STD) (Kaushiki, et al., 2018). The common high-risk HPV viruses are HPV 16 and HPV 18. HeLa belongs to HPV 18 cells.

**SCHEME 1:**
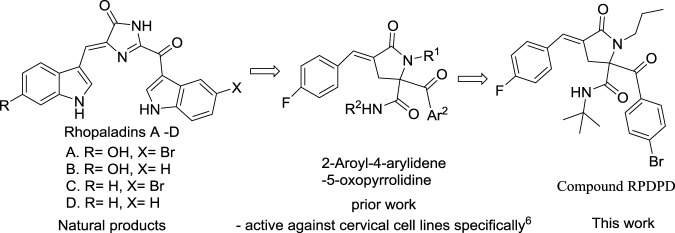
Design strategy of rhopaladins’ analog RPDPD as the target.

**SCHEME 2:**
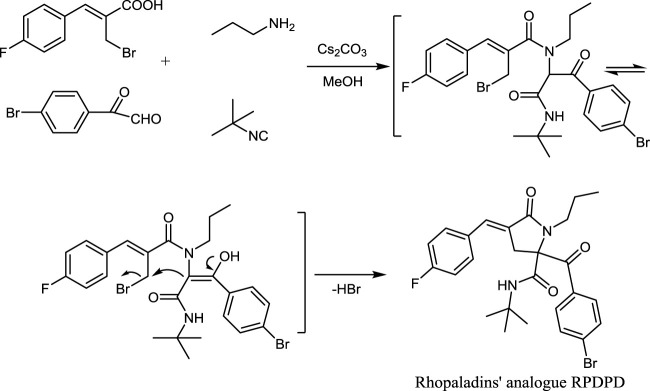
Synthesis of rhopaladins’ analog RPDPD.

The studies have shown that marine alkaloids rhopaladins A–D have significant cytotoxicity to human tumor cell lines. Rhopaladin B has inhibitory activity against CDK4 and c-erbB-2 kinases (IC_50_ of 12.5 and 7.4 μg/ml, respectively) (Hiroyasu et al., 1998; Zhu et al., 2019.). We have previously synthesized rhopaladins’ analogs 2-aroyl-4-arylidene-5-oxopyrrolidine derivatives (see Scheme 1) via a tandem Ugi 4CC/S_N_ cyclization and studied their cytotoxicity to HeLa cells (Wang et al., 2020). According to the existing structure–activity relationship analysis (Wang et al., 2020), in order to obtain a compound with good anti-cervical cancer activity (fluorine-containing) and low hepatotoxicity (bromine-containing), a new rhopaladins’ analog(*E*)-2-(4-bromobenzoyl)-*N*-(*tert*-butyl)-4-(4-fluorobenzylidene) -5-oxo-1-propylpyrrolidine-2-carboxamide (RPDPD for short) of 2-aroyl-4-arylidene-5-oxopyrrolidine derivative was synthesized by a similar method (see Scheme 2). We investigated the cytotoxic and apoptotic effects of compound RPDPD on HeLa cell lines and preliminarily explored the relationship between RPDPD and TIMP3/MMP3 signaling axis.

We used a one-pot Ugi 4CC/S_N_ cyclization method to synthesize 5-oxpyrrolidine -2-carboxamide. The advantages of this synthesis method lie in mild reaction conditions, high yield, and easy availability of raw materials. This new rhopaladins’ analog RPDPD can inhibit the proliferation of the HeLa cell line. Moreover, RPDPD induced HeLa cell apoptosis at early apoptosis. We believed that this synthesis method has potential application prospects in the synthesis of natural products and their analogs, and this compound RPDPD has an important application value as a potential bioactive compound or pharmaceutical.

## Materials and Methods

The melting point was determined by the x-4 model apparatus without correction. The Finnigan trace MS spectrometer was used to detect the compound structure. The infrared absorption of KBr pellets in cm^−1^ was recorded using a PE-983 infrared spectrometer. A Varian mercury 600 spectrometer was used to record NMR in CDCl_3_, which was related to TMS. Elemental analysis was carried out on the Vario-ELIII element analyzer.

The cervical carcinoma cell line HeLa was stemmed from the Chinese Medicine Experiment Center, Affiliated Dongfeng Hospital, Hubei University of Medicine, and maintained in RPMI 1640 medium which contained FBS (10%). The cells were grown in an incubator at 37°C with 5% CO_2_ content.

### One-Pot Synthesis of Rhopaladins’ Analog RPDPD

First, 2-(bromomethyl)-3-phenyl-2-acrylic acid (0.26 g, 1 mmol) was dissolved in methanol (5 ml), and n-propylamine was added (0.06 g, 1 mmol) and was stirred at room temperature for 10 min. Then, 4-bromophenylglyoxal (0.21 g, 1 mmol) and tertbutyl thiocyanate (0.08 g, 1 mmol) were added. The reaction was carried out at room temperature, and the pH value of the reaction system was adjusted to 6 by Cs_2_CO_3_ during the stirring process. After stirring the reaction system for 12 h, the mixture was cooled and filtered with a paper filter. The resulting solid was recrystallized with ether to obtain rhopaladins’ analog RPDPD.

### CCK8 Assay

Seeded in a 96-well plate at a density of 2 × 10^4^ cells/well, the original medium was removed after the cells adhered the next day, and the compound RPDPD (0 μmol/L, 3.125 μmol/L, 6.25 μmol/L, 12.5 μmol/L, 25 μmol/L, 50 μmol/L, and 100 μmol/L) medium was added and cultured for 24, 48, and 72 h, respectively. With DMSO as the control group, cisplatin treatment was used as a positive control, and normal hepatocytes were used for cytotoxicity experiments, and CCK8 (TargetMol, Shanghai, China) was incubated for 2 h. Also, the plate was evaluated in a luminescence microplate reader (BioTEK INC, Biotek MQX200) at 450 nm wavelength. The optical density (OD) values were obtained. The inhibition rate and IC_50_ of each compound on different cells were calculated by GraphPad Prism 8.0.1 software. Cell survival rate (%) = [(A (experimental group)-A (blank)]/[(A (DMSO)-A (blank)]× 100%.

### Apoptotic Assay

The effect of RPDPRH on apoptosis was performed using the Annexin V-FITC/PI Apoptosis kit (MultiSciences, China) following the manufacturer’s protocol. The cells were digested, dispersed, and plated into 6-well plates with 2 × 104 cells/well and treated with RPDPRH (0 μmol/L, 6.25 μmol/L, 12.5 μmol/L, and 25 μmol/L) at various concentrations for 48 h. Upon completion of the treatment, the cells were harvested and washed with cool phosphate buffered solution (PBS, Gibco, United States) (0.01M, pH = 7.4) and resuspended in 500 μl binding buffer. Annexin V-FITC (5 μl) and PI (10 μl) were added to the cell suspension, and then the suspension was incubated at room temperature away from light for 5 min. The cells were analyzed using a flow cytometer (Agilent NovoCyte, China) immediately, and the results of apoptosis were analyzed by Flow J software (Zhu et al., 2020). Set 3 has duplicate holes in each group.

### qRT-PCR Assay

The HeLa cells were treated with the compound **RPDPD** (0 μmol/L, 12.5 μmol/L, 25 μmol/L, and 50 μmol/L) for 48 h. The total RNA was extracted and reverse transcribed into double-stranded cDNA according to the cell processing manual. The amplification procedure for amplifying E6/E7 mRNA was as follows: pre-denaturation at 95°C for 1 min, denaturation at 95°C for 15 s, annealing at 55°C for 15 s, and extension at 72°C for 45 s, a total of 40 cycles. 2^-ΔΔCt^ was used to calculate the relative expression of genes. The primer sets were as follows: E6 F: 5′-TTG​CTT​TTC​GGG​ATT​TAT​GC-3′, R:5′-CAGGACACAGTGGCTTTTGA-3'; E7 F: 5′-GAA​CCG​GAC​AGA​GCC​CAT​TA-3′, R: 5′-AGA​ACA​GAT​GGG​GCA​CAC​AAT-3'. Set 3 has duplicate holes in each group.

### Western Blot Assay

The cells were inoculated into 6-well plates at a cell density of 2 × 10^4^/well, and the supernatant medium was removed after the cells adhered the next day, and 2 ml of the compound RPDPD (0 μmol/L, 12.5 μmol/L, 25 μmol/L, and 50 μmol/L) medium of different concentrations were added to each well and incubated for 48 h. The rabbit anti-TIMP3 antibody (1:1000, Abcam, United Kingdom) and rabbit anti-MMP3 (1:1000, Abcam, United Kingdom) were used for Western blotting. The gray value of protein was analyzed using ImageJ software, and the relative expression of the protein was calculated. Set 3 has duplicate holes in each group.

### Statistical Methods

The statistical software used was SPSS 22.0. The data were expressed as mean ± standard deviation (Mean ± SD), and the data were in line with normal distribution and were compared by one-way ANOVA between groups, and further pairwise comparisons were performed by LSD-t test. *p* < 0.05 indicated that the difference was statistically significant.

## Results and Discussion

### Synthesis of the Compound RPDPD

We used a one-pot Ugi 4CC/S_N_ cyclization reaction to obtained rhopaladins’ analog RPDPD, a white solid, with a yield of 0.42 g, 80% in w/w. The spectral data of RPDPD are as follows. Mp: 197–198°C. 1H NMR (CDCl3, 600 MHz): δ 7.73 (d, J = 8.4 Hz, 2H, Ar-H), 7.61 (d, J = 9.2 Hz, 2H, Ar-H), 7.40 (d, J = 5.2 Hz, 2H, Ar-H), 7.36 (s, 1H, = CH), 7.07 (d, J = 9.2 Hz, 2H, Ar-H), 6.14 (s, 1H, NH), 3.76 (d, J = 17.6 Hz, 1H, CH2a), 3.63–3.55 (m, 1H, NCH2a), 3.28 (d, J = 17.6 Hz, 1H, CH2b), 3.18–3.12 (m, 1H, NCH2b), 1.72–1.51 (m, 2H, CH2), 1.35 (s, 9H, 3CH3), and 0.87 (t, J = 7.2 Hz, 3H, CH3). 13C NMR (CDCl3, 100 MHz): δ 196.1, 169.7, 167.4, 159.4, 132.5, 132.1, 131.5, 130.7, 129.2, 126.1, 124.4, 116.0, 115.8, 74.7, 52.6, 46.1, 35.4, 28.3, 21.3, and 11.6. EI-MS m/z (%): 516 (M++2, 4), 514 (M+, 4), 414 (22), 332 (53), 233 (55), and 183 (100). Anal. Calcd for C26H28BrFN2O3: C, 60.59; H, 5.48; N, 5.44. Found: C, 60.45; H, 5.59; and N, 5.31.

In the experimental process, the reaction conditions were mild, the yield was high, and the raw materials were easy to obtain. The synthetic route of the new fluorine-containing compound RPDPD is outlined in Scheme 2.

### Inhibitory Effects of the Compound RPDPD on HeLa Cell Viability

**FIGURE 1:**
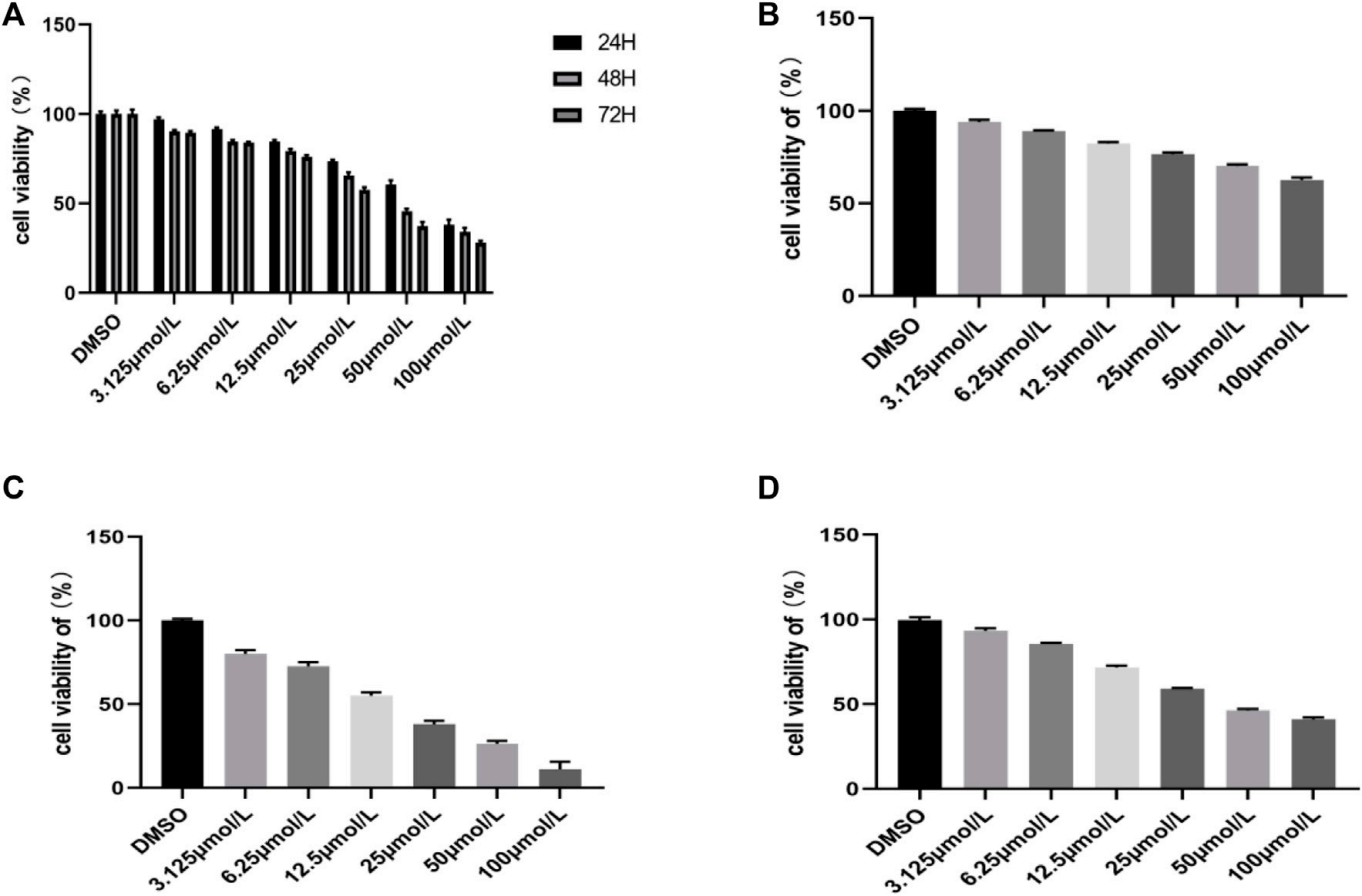
Inhibitory effects of the compound RPDPD on HeLa cells. **(A)** Cell viability of HeLa cells treated with different concentrations of compound RPDPD for 24, 48, and 72 h. **(B)** Cell viability of normal hepatocyte LO2 cells treated with different concentrations of compound RPDPD for 48 h. **(C)** Cell viability of LO2 cells incubated with different concentrations of cisplatin for 48 h. **(D)** Cell viability of HeLa cells incubated with different concentrations of cisplatin for 48 h.

The antiproliferative effect of the compound RPDPD was investigated by CCK8 assay against the cervical carcinoma HeLa tumor cell line. The result exhibited that the compound RPDPD at a varying concentration could inhibit cell growth. We can see that the rate of surviving cells decreased with the increasing drug concentrations and also with time (Figure 1A). The IC_50_ values of compound RPDPD on HeLa cells for 24, 48, and 72 h were 59.56 μmol/L, 39.64 μmol/L, and 24.23 μmol/L, respectively, and the IC_50_ of cisplatin on HeLa cells for 48 h was 17.36 μmol/L. The IC_50_ of compound RPDPD on normal hepatocyte LO2 cells for 48 h was 235.6 μmol/L, but the IC_50_ of cisplatin on normal hepatocyte LO2 cells for 48 h was just 21.32 μmol/L. The results showed that rhopaladins’ analog RPDPD has good anti-cervical cancer activity and low hepatotoxicity, and the inhibition of RPDPD on cell growth was different with varying concentrations, i.e., the higher the concentration, the higher the inhibition rate of the cells. It can be seen from the experimental result that the inhibitory effect of the compound RPDPD on the growth of HeLa cells was exerted in concentration-dependent and time-dependent manners.

### Induction of Apoptosis in HeLa Cells

**FIGURE 2:**
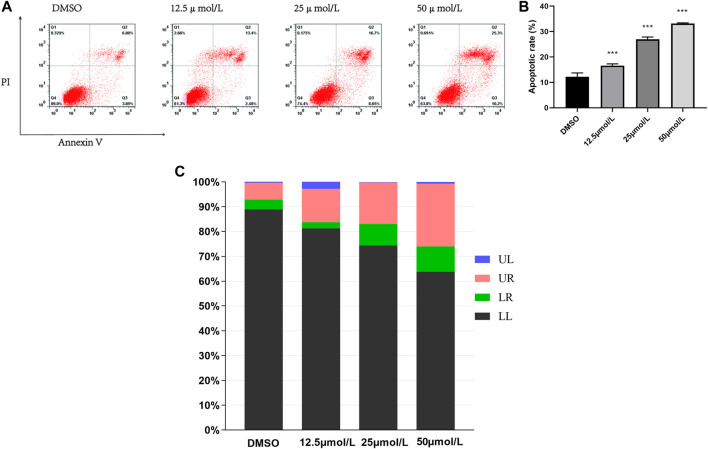
Effect of RPDPD on the apoptosis of HeLa cell. **(A)** FITC/PI double staining analysis of apoptosis induction of HeLa cells after RPDPD treatment for 48 h. Compared with the DMSO control group, **p < 0.01 and ***p < 0.001 (x ± s, *n* = 3). **(B,C)** Living cells (LL), early apoptotic cells (LR), late apoptotic (UR), and necrotic cell/fragment (UL) rate of HeLa were analyzed.

To determine the type of cell death by the compound RPDPD, it was measured by FITC/PI double staining, which can give a differential analysis of apoptosis cells. The results showed that the survival rate of HeLa cells treated with IC_50_ of the compound RPDPD was decreased after 48 h. After the cells were treated with the compound RPDPD, the apoptosis rate of the cells was increased. The FACS flow cytometry analysis showed that HeLa cells were treated with the compound RPDPD at its IC_50_ concentration for 48 h, and the percentage of apoptosis cells was increased in a concentration-dependent manner (Figure 2). There was an increase in percentage of annexin-V-positive cells occurred indicating early apoptosis, and the apoptosis was from 10.67% in control to 35.5% for the compound RPDPD.

### Inhibition of E6 mRNA and E7 mRNA Expressions in HeLa Cells

**FIGURE 3:**
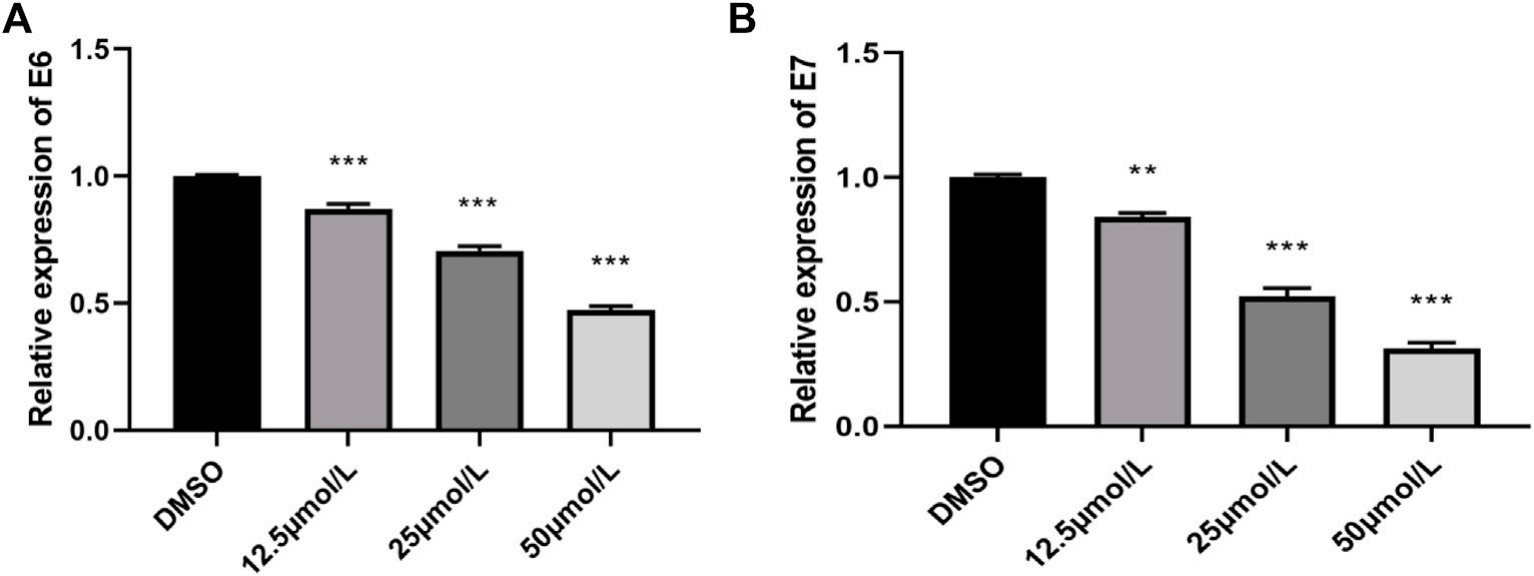
Effect of RPDPD on the expressions of E6 mRNA and E7 mRNA. **(A)** Relative expression levels in E6 mRNA cells. **(B)** Relative expression levels in E7 mRNA cells. *p < 0.05 and **p < 0.01 (x ± s, *n* = 3).

E6/E7 are the main oncogenes of HPV and are involved in various pathological processes in the occurrence and development of cervical cancer. The expression levels of E6 mRNA and E7 mRNA genes were analyzed by qRT-PCR. After RPDPD treatment of HeLa cells for 48 h, the expression levels of E6 mRNA and E7 mRNA genes were significantly decreased in a concentration-dependent manner (*p* < 0.05, Figure 3). The results showed that RPDPD could regulate the expression level of E6/E7 in HeLa cells.

### RPDPD Regulates the TIMP3/MMP3 Signaling Pathway in Cervical Cancer Cells

**FIGURE 4:**
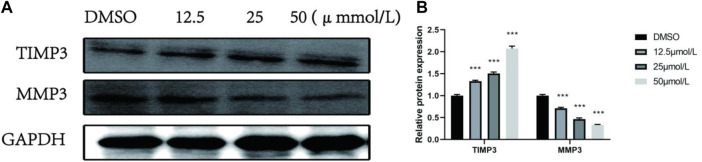
Effect of RPDPRH on the expression levels of TIMP3 and MMP3 proteins. **(A)** Protein expression bands of TIMP3 and MMP3 proteins in HeLa cells. **(B)** Relative protein profiles of TIMP3 and MMP3 proteins in HeLa cells. Compared with the DMSO control group, *p < 0.05 and **p < 0.01 (x ± s, *n* = 3).

To further explore whether the effect of the compound RPDPD on the proliferation, migration, and invasion of cervical cancer cells is mediated by inhibiting TIMP3, the expression levels of TIMP3 and MMP3 were analyzed by Western blot. The Western blot analysis showed that compared with the control group DMSO, the expression of TIMP3 protein in HeLa cells treated with 12.5 μmol/L, 25.0 μmol/L, and 50.0 μmol/L RPDPD was significantly increased in a concentration-dependent manner after the compound RPDPD intervention for 48 h. The expression was significantly downregulated with the increase in drug concentration, and the difference was statistically significant (*p* < 0.05, Figure 4). The results indicated that TIMP3/MMP3 was involved in the tumor suppressor effect of the compound on cervical cancer.

## Discussion and Conclusion

We used a one-pot Ugi 4CC/S_N_ cyclization method to synthesize Rhopaladins’ analog RPDPD which can inhibit proliferation and promote apoptosis in HeLa cell lines. We performed an *in vitro* cell assay for the first time to evaluate the anticancer effect of testing a new compound on cervical cancer.

The integration of high-risk HPV into the DNA of cells and long-term persistent infection are the main causes of cervical cancer, of which HPV 16 is considered to be the main risk factor for squamous cervical lesions and squamous cervical cancer (Wang et al., 2021). In our study, we selected high-risk HPV 16-positive HeLa cells for *in vitro* cell experiments. The results of cell proliferation experiments showed that the compound RPDPD could significantly inhibit the viability of cervical cancer HeLa cells in a time- and dose-dependent manner. However, the toxicity of the compound RPDPD to LO2 in normal hepatocytes was weak. Our further study found that RPDPD not only inhibited cell proliferation but also induced cell apoptosis in a dose-dependent manner. These experimental results suggest that this new fluorine-containing compound RPDPD may have significant anti-cervical cancer effects.

The studies have shown that the oncogenic potential of high-risk HPV 16 genotypes is determined by the E6 and E7 oncogenes. These two viral oncogenes are often co-expressed in cervical cancer cells and regulate the occurrence and maintenance of cervical cancer (Hu et al., 2022). Their open reading frames are directly involved in regulating the growth of cervical cancer cells, play a key role in the proliferation of cervical cancer cells, and are closely related to the apoptosis of cervical cancer cells (Wen et al., 2021; Zhang et al., 2021). In this study, we found that RPDPD could inhibit the expression of E6 mRNA and E7 mRNA, which further proved that the anticancer effect of the compound RPDPD on cervical cancer was closely related to the oncogenes E6 and E7.

The endogenous inhibitors of matrix metalloproteinases (MMPs) play an important role in extracellular matrix (ECM) homeostasis and are involved in tumorigenesis and development, angiogenesis, apoptosis, and tumor metastasis (Deryugina and Quigley, 2006), such as cervical cancer and breast cancer (Duffy et al., 2000; Xie et al., 2016). MMPs are natural factor regulators of tissue inhibitors of metalloproteinases (TIMPs). The studies have shown that TIMP3 can inhibit tumor proliferation and angiogenesis by inhibiting MMP3 protein (Zhang et al., 2018; Sun et al., 2019). The study showed that this study was the first to investigate the effect of RPDPD on the TIMP3/MMP3 signaling axis after treatment of HeLa cells. The results showed that the expression of TIMP3 protein in cells was enhanced, and the expression of MMP3 protein in cells was decreased. It is suggested that the anticancer effect of compound RPDPD may also depend on the TIMP3/MMP3 signaling axis. We demonstrate for the first time that RPDPD may alter cervical cancer progression through TIMP3/MMP3.

In conclusion, our study confirmed that rhopaladins’ analog RPDPD affects the proliferation and apoptosis of cervical cancer cells, inhibits the expression of oncogenes E6 mRNA and E7 mRNA, and its anticancer activity may be related to the TIMP3/MMP3 signaling axis. This study is only the first time to explore its anticancer activity, and we will continue to conduct more in-depth research on the mechanism of the compound RPDPD in the future, so as to provide effective experimental data for the development and application of the compound RPDPD in the clinical treatment of cervical cancer.

## Data Availability

The datasets presented in this study can be found in online repositories. The names of the repository/repositories and accession number(s) can be found in the article/Supplementary Material.
