# Supplementary material for: Synthesis and Anticancer Activity of Rhopaladins’ Analog RPDPD Against the HeLa Human Cervical Cancer Cell Line
Source: Front Chem. 2022 Jun 27;10:921276. doi: 10.3389/fchem.2022.921276 (PMC9277706; doi:10.3389/fchem.2022.921276)

synthesis and anticancer activity of Rhopaladins’ analogue RPDPD against HeLa human cervical cancer cell line

Feng Chen^1,2‡^, Hong-Mei Wang^2‡^, Ling-Qi Kong^2‡^, Qin-Hua Chen^3^, Bin Li^1*^, Li-Na Ke^1*^, Xiao-Hua Zeng^1,2*^

1. Sinopharm Dongfeng General Hospital, Hubei University of Medicine, Shiyan 442008, P. R. of China
2. Hubei Key Laboratory of Wudang Local Chinese Medicine Research, School of Pharmaceutical Sciences, Hubei University of Medicine, Hubei, 442000, P. R. of China
3. Shenzhen Baoan Authentic TCM Therapy Hospital, Shenzhen, Guangdong, 518101, P. R. of China

^‡^ Feng Chen, Hong-Mei Wang and Ling-Qi Kong are the first authors.

**^*^** Bin Li, Li-Na Ke and Xiao-Hua Zeng are the corresponding authors.

Supporting Information

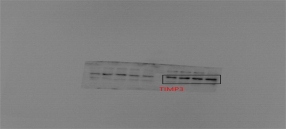

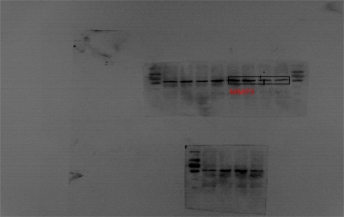

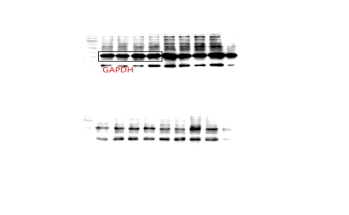

Supplement: Supplementary file 2 [file DataSheet3.docx]
